# Exposure to Endocrine-Disrupting Chemicals during Pregnancy and Weight at 7 Years of Age: A Multi-pollutant Approach

**DOI:** 10.1289/ehp.1409049

**Published:** 2015-05-08

**Authors:** Keren Agay-Shay, David Martinez, Damaskini Valvi, Raquel Garcia-Esteban, Xavier Basagaña, Oliver Robinson, Maribel Casas, Jordi Sunyer, Martine Vrijheid

**Affiliations:** 1 Centre for Research in Environmental Epidemiology (CREAL), Barcelona, Spain; 2 Pompeu Fabra University, Barcelona, Spain; 3 CIBER de Epidemiología y Salud Pública (CIBERESP), Spain; 4 Hospital del Mar Medical Research Institute (IMIM), Barcelona, Spain

## Abstract

**Background:**

Prenatal exposure to endocrine-disrupting chemicals (EDCs) may induce weight gain and obesity in children, but the obesogenic effects of mixtures have not been studied.

**Objective:**

We evaluated the associations between pre- and perinatal biomarker concentrations of 27 EDCs and child weight status at 7 years of age.

**Methods:**

In pregnant women enrolled in a Spanish birth cohort study between 2004 and 2006, we measured the concentrations of 10 phthalate metabolites, bisphenol A, cadmium, arsenic, and lead in two maternal pregnancy urine samples; 6 organochlorine compounds in maternal pregnancy serum; mercury in cord blood; and 6 polybrominated diphenyl ether congeners in colostrum. Among 470 children at 7 years, body mass index (BMI) *z*-scores were calculated, and overweight was defined as BMI > 85th percentile. We estimated associations with EDCs in single-pollutant models and applied principal-component analysis (PCA) on the 27 pollutant concentrations.

**Results:**

In single-pollutant models, HCB (hexachlorobenzene), βHCH (β-hexachlorocyclohexane), and polychlorinated biphenyl (PCB) congeners 138 and 180 were associated with increased child BMI *z*-scores; and HCB, βHCH, PCB-138, and DDE (dichlorodiphenyldichloroethylene) with overweight risk. PCA generated four factors that accounted for 43.4% of the total variance. The organochlorine factor was positively associated with BMI *z*-scores and with overweight (adjusted RR, tertile 3 vs. 1: 2.59; 95% CI: 1.19, 5.63), and these associations were robust to adjustment for other EDCs. Exposure in the second tertile of the phthalate factor was inversely associated with overweight.

**Conclusions:**

Prenatal exposure to organochlorines was positively associated with overweight at age 7 years in our study population. Other EDCs exposures did not confound this association.

**Citation:**

Agay-Shay K, Martinez D, Valvi D, Garcia-Esteban R, Basagaña X, Robinson O, Casas M, Sunyer J, Vrijheid M. 2015. Exposure to endocrine-disrupting chemicals during pregnancy and weight at 7 years of age: a multi-pollutant approach. Environ Health Perspect 123:1030–1037; http://dx.doi.org/10.1289/ehp.1409049

## Introduction

Childhood obesity has increased rapidly since the mid-1980s [World Health Organization/United Nations Environment Programme (WHO/UNEP) 2013]. Greater body mass index (BMI) in childhood is associated with future risk of obesity, cardiovascular disease, certain cancers and a range of other diseases (Baker et al. 2007; Han et al. 2010). There is emerging interest in the possibility that exposure to certain xenobiotic chemicals may be obesogenic (Grün and Blumberg 2009; Holtcamp 2012; La Merrill and Birnbaum 2011) and may change growth patterns and induce weight gain, obesity, and ultimately insulin resistance and type 2 diabetes (La Merrill et al. 2013). It has been suggested that potential effects of obesogens may be strongest when exposure occurs during pregnancy (Huang et al. 2007).

Thus far, the most consistent evidence of obesogenic effects in humans has been reported for gestational tobacco exposure (Oken et al. 2008; Thayer et al. 2012). Over the last two decades, a number of longitudinal epidemiological studies have studied the potential obesogenic effects of prenatal exposures to endocrine-disrupting chemicals (EDCs), and recent literature reviews have summarized these studies (La Merrill and Birnbaum 2011; Lee et al. 2014; Tang-Péronard et al. 2011; Wang et al. 2014; WHO/UNEP 2013). Most epidemiological studies have evaluated potential effects of single persistent organic pollutants, and most have focused on organochlorine compounds, with the most consistent evidence for obesogenic effects thus far reported for dichloro-diphenyldichloroethylene (DDE) (Delvaux et al. 2014; La Merrill and Birnbaum 2011; Lee et al. 2014; Valvi et al. 2014; Warner et al. 2013). Only a few studies have evaluated potential obesogenic effects of prenatal exposure to other groups of EDCs, including bisphenol A (BPA), phthalates, and heavy metals (Delvaux 2014; Gardner et al. 2013; Harley et al. 2013; Tian et al. 2009; Valvi et al. 2013). Recent international expert workshops (e.g., by the U.S. National Toxicology Program) have called for further epidemiological research to establish whether the obesogenic effects seen in animals are supported by evidence in humans (Thayer et al. 2012).

Until now, epidemiological studies on obesogenic effects of *in utero* exposure to EDCs have assessed the risks of single-pollutant exposures. Most human populations are exposed to mixtures of EDCs rather than to a single pollutant, and isolating the potential effects of one EDC exposure from another is difficult when exposures are correlated due to common sources (Sun et al. 2013). Only a few studies have evaluated the health effects of mixtures of EDCs (Braun et al. 2014; Grandjean et al. 2012; Lee et al. 2007, 2010; Lenters et al. 2015; Patel et al. 2010) and none have addressed obesogenic effects. With an increasing number of chemicals now proposed as suspected obesogens, there is a need to identify those most relevant for human obesity risk.

The aim of the current study is to use data on multiple chemical exposures measured in the INMA (“Infancia y Medio Ambiente”—Environment and Childhood) study to evaluate the associations between biomarker concentration of 27 EDCs and child weight status at age 7 years.

## Methods

*Study population*. Data from the Environment and Childhood Project (INMA) in Sabadell (Catalonia, Spain) were used. The study protocol has been described elsewhere (Guxens et al. 2012). Brieﬂy, 657 women were enrolled during 2004–2006, in the first trimester of pregnancy during their first ultrasound visit at the public health center. Women were eligible for participation if they were > 16 years of age, had no communication problems, a singleton pregnancy, and no assisted conception. Questionnaires were administered by trained interviewers during the first (around week 12) and third trimester (around week 32), at delivery, and at 14 months, 4 years, and 7 years after birth to assess maternal and child health status, sociodemographic characteristics, maternal reproductive history, and other characteristics. Ethical approval was obtained from the Clinical Research Ethical Committee of the Municipal Institute of Health Care, and informed consent was obtained from all subjects at each visit.

*Outcome assessment*. Weight (kilograms) and height (centimeters) of the children at approximately 7 years of age (range, 64–95 months) were measured by specially trained nurses; 470 children participated in this follow-up. Child weight and height were measured using standard protocols (without shoes and in light clothing). Age- and sex-specific body mass index (BMI) *z*-scores were calculated based on the WHO standard reference (de Onis et al. 2007, 2009). Overweight was defined as BMI *z*-score (zBMI) ≥ the 85th percentile.

*Chemical exposures*. Our analyses included 27 chemicals, suspected to be EDCs, previously measured in the cohort in biological samples collected during pregnancy or at birth. Specific analytical methods for each group of chemicals are described in their respective reference. Maternal urine was collected in the first and third trimesters of pregnancy and used to measure BPA (Casas et al. 2013), 10 phthalate metabolites (Valvi et al. 2015), and metal concentrations [arsenic (As), lead (Pb) and cadmium (Cd)] (Fort et al. 2014). Maternal blood was collected during the first trimester of pregnancy and used to measure organochlorine pesticides [DDE, hexachlorobenzene (HCB), and β-hexachlorocyclohexane (βHCH)] and polychlorinated biphenyls (PCBs) (Mendez et al. 2011). Cord blood was used to measure total mercury (Hg) concentration (Llop et al. 2012). Maternal colostrum samples collected at the hospital during the first 48–96 hr postpartum were used to measure polybrominated diphenyl ethers (PBDEs) (Gascon et al. 2012). PBDEs were measured in colostrum milk because its higher fat content enabled better detection rates than did cord blood. Further, colostrum levels reflect well the accumulation of maternal exposure during pregnancy (Gascon et al. 2012). To account for urine dilution, the urinary concentrations of phthalate metabolites, BPA, and metals were divided by the urinary concentrations of creatinine (concentrations are expressed in micrograms per gram creatinine for phthalates and BPA, and in nanograms per gram creatinine for metals). For each of these chemicals, the two adjusted urine measurements during pregnancy were averaged because of the high within-person variability characterizing these exposures. The serum concentration of the organochlorine pesticides and the PCBs were lipid-normalized in units of nanograms per gram serum lipid. The cord blood total mercury concentration was expressed in micrograms per liter. The colostrum concentrations of the PBDEs were also lipid-normalized in units of nanograms per gram colostrum lipid.

*Statistical analysis*. All chemical concentrations were log_10_ transformed to obtain normal distributions. Correlations between the transformed concentrations of the 27 EDCs in the original data sets were assessed by computing Pearson correlation coefficients. Linearity of the associations between EDC levels and zBMI were assessed in the original data set using generalized additive models (GAM) (data not shown). Because some of the EDCs demonstrated significant nonlinear associations (*p* for linearity < 0.1) with zBMI, we analyzed all exposure variables in categories defined by tertiles. For zBMI at age 7 years we fitted multiple linear regression models and reported beta coefficients with 95% confidence intervals (CIs). For overweight we fitted generalized linear models with Poisson family, log link, and robust variance to estimate relative risks (RRs) and 95% CIs.

There were missing values in the exposure variables ranging from 3% (organochlorine pesticides and PCBs) to 59.6% (PBDEs) of participants. Only 8.5% of the participants had complete data on all 27 exposure variables. Overall, 28.7% of exposure data values were missing (3,642 data points out of the 12,690 total values for 27 chemicals × 470 children) (see Supplemental Material, Figure S1). Therefore, to evaluate multiple pollutant exposures in one model, we used a multiple imputation approach to impute the missing exposure values (Royston and White 2011). As recommended, to assess the missing at random (MAR) assumption, we first tested whether the likelihood of missing exposure data (missingness) was associated with either of the outcomes (*t*-test for the continues and chi-test for overweight) (data not shown). We then tested whether exposure missingness was associated with known values of confounders and covariates in our data set. To determine which of the variables to include as predictors in the imputation process, we evaluated correlation, *t*-test, or chi-square test with each one of the imputed variables, as appropriate (data not shown). We used imputation models that were more general than the analyses models and included the health outcomes, the variables related to the missingness, and auxiliary variables that were associated with the exposure (Azur et al. 2011; White et al. 2011). We imputed 100 data sets based on the recommendations of Graham (2009). Detailed information regarding the imputation process and a list of the variables used is provided in Supplemental Material, “Description of the Imputation Procedure.” The same approach was applied to impute missing values for the model covariates [missing data ranged from 0.2% (breastfeeding data) to 33.6% (sedentary behavior at age 7 years)].

Potential confounders were selected based on a review of the literature on the determinants of our exposure variables and risk factors for increased BMI (Casas et al. 2013; Forns et al. 2013; Gascon et al. 2012; Llop et al. 2012; Valvi et al. 2013, 2014, 2015). All statistical models were adjusted for the same set of potential confounders: child’s sex (male, female), gestational age (continuous in weeks), birth weight (continuous, in grams), exact age at the time that the outcome was measured (continuous, in months), and maternal country of origin (Spain, non-Spain), maternal age at delivery (continuous, in years), maternal prepregnancy BMI (continuous, in kilograms per meter squared), maternal weight gain during pregnancy (low, recommended, or high) [Institute of Medicine (IOM) 2009], maternal social class [managers, technicians, and associate professionals (nonmanual); other nonmanual workers; and skilled, semiskilled, and unskilled manual workers], breastfeeding duration (less than or more than 16 weeks), and maternal smoking during pregnancy (nonsmoking, any smoking during pregnancy).

We first conducted single-pollutant models for each of the 27 EDCs separately to evaluate the associations between tertiles of exposures and child weight status at age 7 years. Crude and confounder-adjusted models were fitted using complete cases (i.e., removing missing values) and using imputed data sets. Results from multiply imputed data sets were combined using standard multiple imputation rules (White et al. 2011). We evaluated the confounding effect separately for each chemical and co-variable (data not shown).

To evaluate multiple chemical exposures simultaneously, we used principal component analysis. Principal-component analysis (PCA) reduces the number of correlated variables into a smaller number of artificial variables (factors) that capture most of the variance of the original variables while being uncorrelated with each other (Hatcher 1994). This allows the resulting factors to be included within the same model, reducing issues of multicollinearity. To apply PCA on the imputed data set, we first calculated an overall variance–covariance matrix based on within- and between-imputation covariance matrices, and this matrix was used to fit the PCA (Li et al. 1991). We chose to retain four factors based on the scree plot and the number of nontrivial factors (Brown 2009). We applied a Varimax rotation and calculated factor scores for each participant. These four factor scores were categorized into tertiles and included in the regression models. Models included all four factors separately and simultaneously.

Previous studies have suggested that child sex, maternal smoking, maternal BMI, and maternal socioeconomic status may modify the associations between EDCs and later child weight status (Mendez et al. 2011; Thayer et al. 2012; Wang et al. 2014). We evaluated these potential effect modifiers in our study by including in the models the interaction term between the four PCA factors and the possible modifier. We also evaluated the associations between PCA factors and weight status stratified by the categories of the potential effect modifiers. We evaluated the *p*-values for the interaction terms and for the stratified analysis for each of the tertiles. Finally, as a sensitivity analysis we evaluated further adjustment for total daily caloric intake of the child during the last year and hours per day spent in sedentary activities, including time watching television, using computers, and playing video games (three categories: < 1 hr/day during week and < 2 hr/day during weekend; < 1 hr/day during week and 2–3 hr/day during weekend or 1–2 hr/day during week and < 2 hr/day during weekend or 1–2 hr/day during week and 2–3 hr/day during weekend; > 2 hr/day during week or > 3 hr/day during weekend).

Statistical significance was defined as *p*-value < 0.05. All analyses were performed using the statistical package STATA version 12.1 (StataCorp, College Station, TX, USA).

## Results

Our analyses included 470 singleton children with available data on BMI at 7 years of age. Data on 27 EDCs exposure variables and demographic variables are presented in Table 1 and Table 2, respectively. The mean and the geometric mean (GM) concentrations of the 27 EDCs were generally similar in the original and the imputed data set (Table 1). The prevalence of overweight children at follow-up was 31.9% (*n* = 150) (Table 2). Mothers were predominantly of Spanish origin (91.7%), from lower socioeconomic class (44.5%), with a high prevalence of higher than recommended weight gain during pregnancy (38.9%), and a high smoking rate during pregnancy (27.2%) (Table 2). There were minor differences in the imputed data set distributions compared with the original data set (Table 2). Correlation coefficients were generally weaker between EDCs from different chemical groups compared with those within groups (see Supplemental Material, Figure S2).

**Table 1:** Concentrations and percentage of quantifiable and missing samples for the 27 EDCs in the original data set and the imputed data set (*n* = 470).

EDCs	Complete case, original data set						Imputed data set	
	< LOD (*n*)	Missing (*n*)	Minimum	Maximum	Mean (95% CI)	GM	Mean (95% CI)	GM
MEP^*a*^	0	110	34	9379.9	605.4 (525.2, 685.7)	379.5	597.8 (523.6, 672.1)	376.1
MnBP^*a*^	0	110	5.8	835.7	46.1 (39.2, 53)	32.4	45.6 (39.8, 51.4)	32.7
MiBP^*a*^	0	110	5.1	334.2	41.5 (37.4, 45.5)	32.6	41.1 (37.5, 44.7)	32.6
MBzP^*a*^	0	110	1.5	405.1	19.1 (15.7, 22.5)	12.5	18.8 (16, 21.7)	12.6
7OHMMeOP^*a*^	81	122	0.4	343.5	3.4 (1.5, 5.4)	1.7	3.2 (1.7, 4.6)	1.8
MECPP^*a*^	0	110	7.7	718.9	51.6 (46.1, 57.1)	40.8	50.9 (46.1, 55.6)	40.6
MEHHP^*a*^	0	110	5.3	503.4	38.2 (33.5, 42.9)	28.6	37.6 (33.6, 41.5)	28.6
MEOHP^*a*^	0	110	4.1	378.3	27.8 (24.5, 31.1)	21.5	27.4 (24.6, 30.2)	21.4
MEHP^*a*^	0	110	1.8	266.9	14.6 (12.8, 16.3)	11.2	14.5 (13, 16)	11.2
MCMHP^*a*^	0	182	14.4	1086.5	58.3 (49.7, 66.9)	45.9	56.1 (50, 62.1)	45.3
BPA^*a*^	0	97	0.3	69.4	3.7 (3.1, 4.2)	2.7	3.6 (3.2, 4.1)	2.7
Cd^*b*^	23	188	0.1	5.9	0.7 (0.6, 0.8)	0.6	0.7 (0.6, 0.8)	0.6
As^*b*^	0	99	2.9	702.2	65.5 (57.7, 73.3)	42.8	66.1 (58.6, 73.6)	43
Pb^*b*^	3	190	0.5	25.2	5.1 (4.6, 5.6)	4.2	5.3 (4.9, 5.8)	4.4
Hg^*c*^	24	120	1.4	60	8.1 (7.5, 8.8)	6.3	8.2 (7.6, 8.9)	6.4
DDE^*d*^	1	14	7.7	17263.4	236.4 (152.3, 320.5)	126.3	235.5 (153.8, 317.2)	126.8
HCB^*d*^	34	14	4.5	293	51.9 (48.1, 55.7)	38.5	51.9 (48.1, 55.7)	38.4
βHCH^*d*^	45	14	4.4	497.6	41.2 (37.8, 44.6)	31.3	41.2 (37.8, 44.6)	31.3
PCB-138^*d*^	100	14	4.5	98.1	20.4 (19.2, 21.5)	16.9	20.3 (19.2, 21.5)	16.8
PCB-153^*d*^	33	14	4.5	154.9	37.3 (35.4, 39.2)	31.5	37.3 (35.4, 39.2)	31.4
PCB-180^*d*^	75	14	3.9	119.7	25.2 (23.8, 26.6)	20.7	25.1 (23.7, 26.5)	20.6
PBDE-47^*e*^	61	280	0.1	15	1 (0.7, 1.2)	0.5	1 (0.8, 1.1)	0.5
PBDE-99^*e*^	59	280	0	8.9	0.6 (0.4, 0.7)	0.3	0.6 (0.5, 0.7)	0.3
PBDE-100^*e*^	39	280	0	3.2	0.4 (0.3, 0.4)	0.2	0.4 (0.3, 0.4)	0.2
PBDE-153^*e*^	24	280	0.1	12.2	1 (0.8, 1.1)	0.7	1 (0.8, 1.2)	0.7
PBDE-154^*e*^	25	280	0.1	12.2	1 (0.8, 1.1)	0.7	1 (0.8, 1.2)	0.7
PBDE-209^*e*^	37	280	0	5.3	1.1 (1.0, 1.3)	0.8	1.1 (1, 1.2)	0.8
Abbreviations: As, arsenic; βHCH, β-hexachlorocyclohexane; BPA, bisphenol A; Cd, cadmium; CI, confidence interval; DDE, dichlorodiphenyldichloroethylene; EDCs, endocrine-disrupting chemicals; GM, geometric mean; HCB, hexachloro­benzene; Hg, mercury; LOD, limit of detection; MBzP, monobenzyl phthalate; MEHHP, mono(2-ethyl-5-hydroxyhexyl) phthalate; MEHP, mono(2-ethylhexyl) phthalate; MEOHP, mono(2-ethyl-5-oxohexyl) phthalate; MEP, monoethyl phthalate; MiBP, monoisobutyl phthalate; MnBP, mono-*n*-butyl phthalate; Pb, lead; PBDEs, polybrominated diphenyl ethers; PCBs, polychlorinated biphenyls; MCMHP, mono(2-carboxyhexyl) phthalate; MECPP, mono(2-ethyl-5-carboxypentyl) phthalate; 7OHMMeOP, mono(4-methyl-7-hydroxyoctyl) phthalate. ^***a***^First- and third-trimester urine (μg/g creatinine). ^***b***^First- and third-trimester urine (ng/g creatinine). ^***c***^Cord blood (μg/L). ^***d***^First-trimester serum (ng/g serum lipid). ^***e***^Colostrum (ng/g colostrum lipid).								

**Table 2:** Characteristics (mean or percent) of 470 children and their mothers at child’s age 7 years, in the original and imputed data sets.*^a^*

Characteristic	Missing (*n*)	Original data set mean or percent (95% CI)	Imputed data set mean or percent (95% CI)
Child characteristics
zBMI at age 7 years (mean)	0	0.7 (0.6, 0.8)	0.7 (0.6, 0.8)
Overweight at age 7 years (%)	0	31.9 (27.7, 36.1)	31.9 (27.7, 36.1)
Female sex (%)	0	51.3 (46.7, 55.8)	51.3 (46.7, 55.8)
Gestational age (weeks) (mean)	7	39.7 (39.6, 39.9)	39.7 (39.6, 39.9)
Birth weight (g) (mean)	0	3261.9 (3223.8, 3299.9)	3261.9 (3223.8, 3299.9)
Exact age at 7 years (months) (mean)	0	81.8 (81.4, 82.2)	81.8 (81.4, 82.2)
Any breastfeeding > 16 weeks (%)	2	68.4 (64.2, 72.6)	68.3 (64, 72.5)
Child time spent watching TV or playing video games (hr/day)	158
< 1 during week and < 2 during weekend		18.3 (14, 22.6)	15.8 (12, 19.7)
< 1 during week and 2–3 during weekend, 1–2 during week and < 2 during weekend, 1–2 during week and 2–3 during weekend		43.9 (38.4, 49.5)	45.4 (40, 50.8)
> 2 during week or > 3 during weekend		37.8 (32.4, 43.2)	38.8 (33.7, 44)
Child daily total caloric intake	58	1635.3 (1601.9, 1668.9)	1641.9 (1608.2, 1675.6)
Maternal characteristics
Age at delivery (years) (mean)	1	31.8 (31.5, 32.2)	31.8 (31.5, 32.2)
Prepregnancy BMI (kg/m^2^) (mean)	0	23.8 (23.4, 24.2)	23.8 (23.4, 24.2)
Maternal weight gain during pregnancy (IOM) (%)	15
Recommended		42.9 (38.3, 47.4)	43 (38.5, 47.6)
Lower than recommended		18.2 (14.7, 21.8)	18.3 (14.8, 21.9)
Higher than recommended		38.9 (34.4, 43.4)	38.7 (34.2, 43.1)
Country of origin, Spain (%)	3	91.7 (89.1, 94.2)	91.6 (89.1, 94.2)
Social class (ISCO-88 code) (%)	0
Professionals and managers (I, II)		22.8 (19, 26.6)	22.8 (19, 26.6)
Other nonmanuals (III)		32.8 (28.5, 37)	32.8 (28.5, 37)
Skilled, semiskilled, and unskilled manual (IV, V)		44.5 (40, 49)	44.5 (40, 49)
Smoking during pregnancy (%)	6	27.2 (23.1, 31.2)	27.3 (23.2, 31.3)
Daily total caloric intake during pregnancy (kcal/day)	0	1641.9 (1608.2, 1675.6)	1641.9 (1608.2, 1675.6)
ISCO-88, International Standard Classification of Occupations. ^***a***^Imputation model for each variable was more general than the analyses models and included the health outcomes, the variables related to the missingness and auxiliary variables that were associated with the exposure (100 data sets were imputed). Detailed information regarding the imputation process and a list of the variables used is provided in Supplemental Material, “Description of the Imputation Procedure.”			

**Figure 1:**
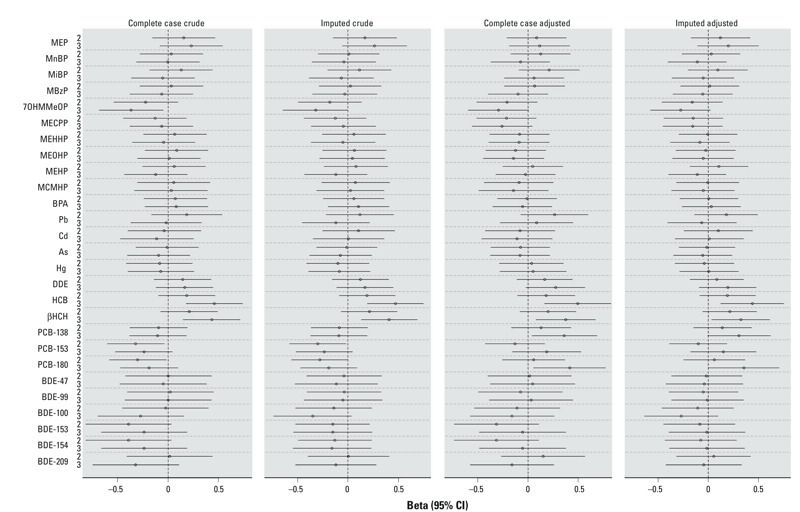
Crude and adjusted associations [β coefficient (95% CI)] between maternal exposure to tertiles of 27 EDCs and child zBMI at age 7 years, single- pollutant models, for complete case and imputed data (*n *= 470). Abbreviations: As, arsenic; BDE, polybrominated diphenyl ethers congeners; βHCH, β-hexachlorocyclohexane; BPA, bisphenol A; Cd, cadmium; CI, confidence interval; DDE, dichlorodiphenyldichloroethylene; EDCs, endocrine-disrupting chemicals; HCB, hexachlorobenzene; Hg, Mercury; MBzP, monobenzyl phthalate; MEHHP, mono(2-ethyl-5-hydroxyhexyl) phthalate; MEHP, mono(2-ethylhexyl) phthalate; MEOHP, mono(2-ethyl-5-oxohexyl) phthalate; MEP, mono-ethyl phthalate; MiBP, monoisobutyl phthalate; MnBP, mono-*n*-butyl phthalate; Pb, lead; MCMHP, mono(2-carboxyhexyl) phthalate; MECPP, mono(2-ethyl-5-carboxypentyl) phthalate; 7OHMMeOP, mono(4-methyl-7-hydroxyoctyl) phthalate; PCBs, polychlorinated biphenyl congeners; zBMI, body mass index *z*-score.

Single-pollutant, complete-case, adjusted models for the 27 EDCs exposures showed a statistically significant increase in zBMI with increasing exposure to HCB [adjusted (adj) β tertile 3 vs. 1: 0.49; 95% CI: 0.16, 0.82], βHCH (adj β tertile 3 vs. 1: 0.37; 95% CI: 0.08, 0.82), PCB-138 (adj β tertile 3 vs. 1: 0.36; 95% CI: 0.04, 0.68), and PCB-180 (adj β tertile 3 vs. 1: 0.41; 95% CI: 0.05, 0.77) (Figure 1; see also Supplemental Material, Table S1). For DDE, tertile 3 estimates were increased compared with tertile 1 and nearly reached statistical significance (adj β tertile 3 vs. 1: 0.27; 95% CI: –0.02, 0.56). For 7OHMMeOP [mono(4-methyl-7-hydroxyoctyl) phthalate], tertile 3 estimates were decreased compared to tertile 1 and nearly reached statistical significance (adj β tertile 3 vs. 1: –0.29; 95% CI: –0.59, 0.01). Certain phthalates and certain PBDEs showed nonsignificant negative associations. For example, for PBDE-53 and PBDE-54 tertile 2 estimates were decreased compared with tertile 1 (adj β tertile 2 vs. 1: –0.31; 95% CI: –0.73, 0.11) (Figure 1; see also Supplemental Material, Table S1).

Adjusted estimates based on the imputed data set were very similar to those based on the complete case analyses (Figure 1; see also Supplemental Material, Table S1). Adjustment for the covariates in the single-pollutant models had some small effects on the point estimates for most chemical exposures (Figure 1; see also Supplemental Material, Table S1). However, for the PCBs, changes in the direction of the estimate associations were observed. For example, for PCB-180 tertile 3 estimates compared with tertile 1 were changed from negative to positive associations between the crude and adjusted models (crude β tertile 3 vs. 1: –0.19; 95% CI: –0.47, 0.09; adj β tertile 3 vs. 1: 0.41; 95% CI: 0.05, 0.77). These changes were attributable mainly to adjustment for maternal prepregnancy BMI.

Generally, results for single-pollutant, complete-case, adjusted models using overweight as the outcome measure were similar in direction to results using zBMI (see Supplemental Material, Figure S3). For HCB, βHCH, and PCB-138 associations with overweight were statistically significant in the third tertiles of exposure (adj RR tertile 3 vs. 1: 2.17; 95% CI: 1.08, 4.38; adj RR tertile 3 vs. 1: 1.94; 95% CI: 1.04, 3.61; and adj RR tertile 3 vs. 1: 2.14; 95% CI: 1.05, 4.35; respectively). For PCB-180, associations with overweight nearly reached statistical significance in the third tertiles of exposure (adj RR tertile 3 vs. 1: 2.15; 95% CI: 0.99, 4.70). For DDE associations with overweight were statistically significant in both the second (adj RR tertile 2 vs. 1: 2.31; 95% CI: 1.26, 4.24) and the third tertiles of exposure (adj RR tertile 3 vs. 1: 2.21; 95% CI: 1.17, 4.15). For MECPP [mono(2-ethyl-5-carboxypentyl) phthalate], inverse associations with overweight were statistically significant in both the second (adj RR tertile 2 vs. 1: 0.49; 95% CI: 0.24, 0.98) and the third tertiles of exposure (adj RR tertile 3 vs. 1: 0.43; 95% CI: 0.22, 0.85). The RRs estimates were similar in the imputed data set (see Supplemental Material, Figure S3).

Using PCA on the 27 EDCs, we generated four factors that accounted for 43.4% of the total variance inherent in the data. Detailed information regarding the factor loading is presented in the Supplemental Material, Table S2. The PCA Varimax rotation was as follows: Factor 1 was highly loaded with PBDEs (explained variation, 15.0%): PBDE-47 (0.55), PBDE-99 (0.55), PBDE-100 (0.37), PBDE-153 (0.31), PBDE-154 (0.31), and PBDE-209 (0.23); factor 2 was highly loaded with phthalates (explained variation 12.7%): MnBP (mono-*n*-butyl phthalate; 0.25), MiBP (monoisobutyl phthalate; 0.21), MBzP (monobenzyl phthalate; 0.28), 7OHMMeOP (0.31), MECPP (0.37), MEHHP [mono(2-ethyl-5-hydroxyhexyl) phthalate; 0.41], MEOHP [mono(2-ethyl-5-oxohexyl) phthalate; 0.39], MEHP [mono(2-ethylhexyl) phthalate; 0.32], and MCMHP [mono(2-carboxymethylhexyl) phthalate; 0.31]; factor 3 was highly loaded with organochlorines (explained variation 9.6%): DDE (0.24), HCB (0.51), βHCH (0.45), PCB-138 (0.36), PCB-153 (0.38), and PCB-180 (0.37); and factor 4 (explained variation 6.1%) was highly loaded with MEP (monoethyl phthalate; 0.32), As (0.67), Hg (0.31), BPA(0.23), PBDE-153 (0.23), PBDE-154 (0.21), DDE (–0.24), and βHCH (–0.21).

In the model that simultaneously included all four factors, exposure to the highest tertile compared with the lowest tertile of the organochlorine factor (factor 3) was associated with significant increase in the zBMI of 0.37 (95% CI: 0.03, 0.72) and with an increase in the RRs of overweight of 2.59 (95% CI: 1.19, 5.63). In tertile 2, zBMI and overweight demonstrated a nonsignificant increase (adj β tertile 2 vs. 1: 0.12; 95% CI: –0.19, 0.43; adj RRs tertile 2 vs. 1: 1.86; 95% CI: 0.92, 3.76) (Table 3). Exposure to the phthalate factor (factor 2) showed a decrease in the RRs for overweight of 0.49 (95% CI: 0.25, 0.96) in tertile 2 and nonsignificant negative associations in tertile 3 compared with tertile 1 of 0.63 (95% CI: 0.33, 1.19). Similar, nonsignificant negative associations were observed with zBMI for exposure to the phthalate factor (factor 2) in tertile 3 and tertile 2 compared with tertile 1. Exposure to the PBDE factor (factor 1) showed a nonsignificant decrease in the RRs for overweight (adj RRs tertile 2 vs. 1: 0.61; 95% CI: 0.28, 1.34; adj RRs tertile 3 vs. 1: 0.54; 95% CI: 0.25, 1.17). Similar, nonsignificant negative associations with zBMI were observed for exposure to the PBDE factor (factor 1) in tertile 3 and tertile 2 compared to tertile 1 (Table 3). Results from models including each single factor separately were similar to the results of the models simultaneously adjusting for all factors (Table 3).

**Table 3:** Association between maternal exposure to tertiles of the four factors from principal-component analysis and BMI *z*-score or overweight at age 7 years based on single- and multiple-factor models (imputed data, *n *= 470).

Exposure	zBMI		Overweight	
	Single-factor model β (95% CI)	Multiple-factor model β (95% CI)	Single-factor model RR (95% CI)	Multiple-factor model RR (95% CI)
Factor 1: PBDEs^*a*^
1	Reference	Reference	Reference	Reference
2	–0.12 (–0.47, 0.23)	–0.15 (–0.50, 0.21)	0.69 (0.33, 1.44)	0.61 (0.28, 1.34)
3	–0.14 (–0.48, 0.20)	–0.19 (–0.54, 0.16)	0.59 (0.29, 1.22)	0.54 (0.25, 1.17)
Factor 2: phthalates^*b*^
1	Reference	Reference	Reference	Reference
2	–0.15 (–0.44, 0.15)	–0.15 (–0.45, 0.15)	0.49 (0.26, 0.94)	0.49 (0.25, 0.96)
3	–0.10 (–0.38, 0.18)	–0.13 (–0.42, 0.17)	0.66 (0.36, 1.19)	0.63 (0.33, 1.19)
Factor 3: organochlorine^*c*^
1	Reference	Reference	Reference	Reference
2	0.10 (–0.20, 0.41)	0.12 (–0.19, 0.43)	1.68 (0.85, 3.32)	1.86 (0.92, 3.76)
3	0.34 (0.0, 0.68)	0.37 (0.03, 0.72)	2.17 (1.05, 4.49)	2.59 (1.19, 5.63)
Factor 4: MEP, As, Hg, BPA, PBDE‑153, PBDE‑154^*d*^
1	Reference	Reference	Reference	Reference
2	–0.05 (–0.36, 0.26)	–0.04 (–0.36, 0.28)	0.96 (0.48, 1.93)	0.99 (0.47, 2.11)
3	0.04 (–0.27, 0.35)	0.06 (–0.27, 0.38)	0.89 (0.47, 1.67)	0.95 (0.47, 1.90)
All models were adjusted for child’s sex, gestational age, birth weight, exact age at the time that the outcome was measured (months), maternal country of origin, maternal age at delivery, maternal prepregnancy BMI, maternal weight gain during pregnancy, maternal social class, breastfeeding duration and maternal smoking during pregnancy. Multiple- factor models were also adjusted for all four factors in a single model. ^***a***^Factor 1 loaded with PBDEs: BDE-47, BDE-99, BDE-100, BDE-153, BDE-154, BDE-209. ^***b***^Factor 2 loaded with phthalates: MnBP, MiBP, MBzP, 7OHMMeOP, MECPP, MEHHP, MEOHP, MEHP, MCMHP. ^***c***^Factor 3 loaded with organochlorines: DDE, HCB, βHCH, PCB-138, PCB-153, PCB-180. ^***d***^Factor 4 loaded with MEP, As, Hg, BPA, PBDE-153, PBDE-154, DDE, and βHCH; had negative loading values.				

There was no evidence for modification of the association between factors of exposure and child weight status by child’s sex, maternal prepregnancy BMI, maternal socioeconomic class and maternal smoking status (*p*-values for interaction > 0.2; data not shown). The results of the sensitivity analyses further adjusting for child total daily caloric intake and child sedentary behavior during the last year were also not different from our main analyses (data not shown).

## Discussion

To our knowledge, this study is the first to investigate the combined effects of pre- and perinatal exposure to 27 suspected EDCs on child weight status, using a multi-pollutant approach. Maternal serum concentrations of organochlorine compounds were related to weight status at age 7 years in single-pollutant and PCA, and these associations were robust to the adjustment for other EDCs exposures. A factor reflecting combined exposure to multiple phthalate metabolites showed weak evidence for an association with reduced BMI. Exposure to other EDCs, whether in single-pollutant or combined multi-pollutant analyses, showed no evidence for an association with child weight status.

Our results are largely consistent with the existing literature: We observed an increase in zBMI with increased prenatal exposure to the organochlorine compounds HCB, βHCH, PCB-138, and PCB-180; an increase in overweight at age 7 years with exposure to HCB, βHCH, PCB-138, and DDE; and increased *z*-scores and overweight with increased combined exposures to the PCA factor combining these compounds. Our findings for HCB are consistent with those of some previous studies (Smink et al. 2008; Valvi et al. 2014), but others did not observe any associations (Delvaux et al. 2014; Mendez et al. 2011; Verhulst et al. 2009). Only one study evaluated prenatal exposure to βHCH, and associations were positive but not statistically significant (Mendez et al. 2011). Results of the 13 studies that investigated PCBs were not consistent, and associations in some studies were shown to be modified by sex (Blanck et al. 2002; Delvaux et al. 2014; Gladen et al. 2000; Hertz-Picciotto et al. 2005; Jacobson et al. 1990; Karmaus et al. 2009; Lamb et al. 2006; Mendez et al. 2011; Patandin et al. 1998; Tang-Péronard et al. 2014; Valvi et al. 2012, 2014; Verhulst et al. 2009). Consistent with our study, a number of previous longitudinal studies have reported associations between prenatal DDE exposure and increased BMI and overweight risk (Cupul-Uicab et al. 2010; Delvaux et al. 2014; Gladen et al. 2000, 2004; Karmaus et al. 2009; Mendez et al. 2011; Valvi et al. 2012, 2014; Verhulst et al. 2009; Warner et al. 2013). In our study, we did not find significant associations with BPA, cadmium, lead, mercury, arsenic, and PBDE exposure with child weight status later in life. Only two longitudinal studies evaluated the effect of prenatal BPA exposure on child BMI: Harley et al. (2013) found no significant associations, and Valvi et al. (2014) reported a weak but nonsignificant positive association with BMI. For cadmium, Gardner et al. (2013) reported an inverse association with child weight, whereas two other studies reported nonsignificant positive associations with child weight (Delvaux et al. 2014; Tian et al. 2009). For lead, two studies evaluated the associations with weight, and results were nonsignificant (Tian et al. 2009; Gardner et al. 2013). One study has reported an association with lower weight with arsenic exposure (Gardner et al. 2013). In the INMA cohort, using the same data set, we have recently shown that the sum of high-molecular-weight phthalates was inversely associated with BMI in boys but not in girls (Valvi et al. 2015). The results of our present PCA analysis showing a lower BMI for the second-tertile exposure compared with the first are consistent with this; however, we did not observe evidence of effect modification by sex (data not shown). To the best of our knowledge, the present study is the first longitudinal analysis of childhood body weight and prenatal exposure to PBDEs and mercury. For these chemicals there is some evidence for obesogenic effects from previous cross-sectional and toxicological studies (La Merrill and Birnbaum 2011; Tang-Péronard et al. 2011; Wang et al. 2014; WHO/UNEP 2013).

Only a few studies related to EDCs exposures and health outcomes have used a multi-pollutant approach (Braun et al. 2014; Grandjean et al. 2012; Lee et al. 2007, 2010; Lenters et al. 2015; Patel et al. 2010). To our knowledge, there is no epidemiological study on the association between gestational exposure to multiple EDCs and child weight status. Generally, the main strength of multi-pollutant approaches is the ability to evaluate associations for many exposures simultaneously, which may differ from summing the separate effects of each chemical from single-pollutant models (Billionnet et al. 2012; Mauderly et al. 2010; Sun et al. 2013). We used PCA to evaluate multi-pollutant exposures, and estimated a statistically significant association between an organochlorine-loaded factor and child weight status (zBMI and overweight) at age 7 years. PCA is widely used in dietary studies to describe dietary patterns and to study the associations of these patterns (factors) with health outcome (Kant 2004). To our knowledge, in studies of environmental pollutant exposures, PCA has been used in only two previous studies of air pollution and respiratory outcomes (Arif and Shah 2007; Qian et al. 2004). The use of PCA has some advantages. First, it minimizes the multi-collinearity problem because the loaded factors are orthogonal and the correlated variables are blocked within their factor. Second, each factor represents a weighted combination of the individual EDCs that it represents. Because the relationship between the exposures and outcome is not accounted for in the generation of the factors, the same factors can be used to evaluate associations with other health outcomes. However, this may also be a limitation in the specific case where the single pollutant that is most related to the health outcomes is not represented in the factors that created the PCA (Sun et al. 2013). Our PCA produced four factors of which three were clearly defined by specific exposure groups (PBDEs, phthalates, organochlorines). The fourth factor, though, was a mix of chemicals from different exposure groups, reflecting a pattern of higher MEP, As, Hg, BPA, and PBDE exposure and lower DDE and βHCH exposure. The reasons for this patterning are not clear and require further evaluation. A further limitation of the PCA approach is that it does not explicitly allow for the evaluation of interactions between chemicals (such as synergism, antagonism, or inhibition). However, the PCA method enabled us to evaluate the integrated effect of exposure to an organochlorine mixture, and this can be considered a useful complementary approach to identifying the individual effects of single chemicals. Future studies may consider statistical methods that account for the association between exposure and health outcomes in the selection of exposure variables, such as semi-Bayesian models (Braun et al. 2014) and penalized regression methods (Lenters et al. 2015), or consider summing the EDCs according to their biological activity and toxicological aspects that are relevant to obesity etiology (Lee et al. 2010). Also, approaches that allow for interactions between chemicals to be tested require further consideration; here, boosted regression tree techniques have been suggested as a useful approach (Lampa et al. 2014), but their interpretation can be complex. Multiple comparison issues are important in the interpretation of multi-pollutant studies. In our study we did not adjust statistically for multiple testing in the single-pollutant models. Instead, we complemented the single-pollutant models with a PCA approach to reduce the dimension of the data, taking into account the correlations between exposure variables. This resulted in a test of four PCA factors only. Furthermore, instead of applying an overly conservative multiple comparison adjustment, we draw our conclusions based on the consistency of results between the single-pollutant and PCA approach (Perneger 1998; Rothman 1990).

In our study there were missing values for many exposure variables. In epidemiological studies, the most common strategy for dealing with missing data is a complete-case analysis where participants with missing data on any variable are excluded from the analyses. This may introduce selection bias because the analysis sample no longer retains proportions of the original population. In addition, dropping observed values on some variables for a subject with missing values on other variables may lead to a loss of information. This issue is a special concern when dealing with many exposures (Basagaña et al. 2013). We used multiple imputation to address the missing data problem. This technique provides valid results under the MAR assumption (Desai et al. 2011a, 2011b; Heitjan 2011; Sterne et al. 2009; White et al. 2011). Given the large number of important variables included in the imputation process, we believe it is a fair assumption to consider that missingness was probably unrelated to the actual (unmeasured) exposure after conditioning on these covariates, and thus the MAR assumption held. Multiple imputation of missing values is still not common practice in epidemiological studies (Klebanoff and Cole 2008), and future studies evaluating multiple exposures may consider the technique to decrease bias and inaccuracy of estimates. We fitted the single-pollutant models separately in the original and imputed data sets and found no differences in effect estimates. This supported the evaluation of multi-pollutant models in the imputed data set.

In multi-pollutant studies, it is likely that different exposures are measured with different degrees of accuracy. Different levels of exposure misclassification may lead to different levels of bias in the effect estimates, limiting to some extent the conclusions that can be drawn from a comparison of effect sizes for different pollutants. In our study, for example, serum concentrations of organochlorines give a reliable estimate of long-term exposure because their half-lives are on the order of several years, whereas urine concentrations of nonpersistent EDCs (e.g., the phthalates and BPA) give an estimate of very short-term exposure because their half-lives are on the order of hours or days (WHO/UNEP 2013). Although we averaged the concentrations of this latter group of chemicals over two points during the pregnancy, thus giving a better approximation of average exposure, nondifferential exposure misclassification may occur.

A strength of this study is that we were able to evaluate an extensive list of potential confounders, including sociodemographic, dietary, and physical activity–related factors in the mothers and children. Because our effect estimates in the single-pollutant analyses generally did not change between the crude and the adjusted models after inclusion of child and maternal characteristics, we conclude that these factors did not in fact have a large overall confounding effect and are unlikely to explain our findings. Even so, for each of the PCBs, maternal prepregnancy BMI changed the direction of effect estimates. This has been noted before in a previous analysis of our study population in earlier childhood (Mendez et al. 2011). We further examined the effect of several potential effect modifiers—child sex, maternal smoking, maternal BMI, and maternal socioeconomic status—because these have previously been reported to modify the associations between prenatal EDC exposure and later child weight status (Mendez et al. 2011; Thayer et al. 2012; Wang et al. 2014). However, we did not find any evidence for these variables to modify the effect of the single pollutants or the PCA-derived factors, and our results were relatively consistent in the different strata defined by these variables: boys and girls, smokers and nonsmokers, higher and lower social classes, and normal and overweight/obese mothers.

A limitation of this study is that we did not have data for other potential obesogens such as perfluorinated chemicals, polychlorinated dibenzodioxins (PCDDs), or organotins (La Merrill and Birnbaum 2011; Tang-Péronard et al. 2011; Wang et al. 2014; WHO/UNEP 2013). Evidence for obesogenic effects of these compounds comes mainly from experimental studies, with human studies so far available only for perfluorinated chemicals (Andersen et al. 2013; Halldorsson et al. 2012; Maisonet et al. 2012). In our study we focused only on the potential obesogens that were already measured previously in our population. Although it would have given a more complete comparison of obesogenic effects of different chemicals, because no confounding effect was observed between the factors in our analysis, we consider it unlikely that the inclusion of data on these other groups of chemicals would have changed the current results.

## Conclusions

In our study population, prenatal exposure to organochlorine compounds was associated with overweight in children at 7 years of age, and this association did not appear to be confounded by other EDC exposures. We recommend that other epidemiological studies consider multi-pollutant approaches together with single-pollutant approaches, especially when dealing with correlated exposures. Our findings for organochlorine exposures highlight the fact that it is difficult, if not impossible, to disentangle individual associations of highly correlated exposures; therefore public health action is needed to reduce exposure to mixtures of organochlorines as a whole.

## Supplemental Material

(1.2 MB) PDF
