# Supplementary material for: Exposure to Endocrine-Disrupting Chemicals during Pregnancy and Weight at 7 Years of Age: A Multi-pollutant Approach
Source: Environ Health Perspect. 2015 May 8;123(10):1030–7. doi: 10.1289/ehp.1409049 (PMC4590760; doi:10.1289/ehp.1409049)
Supplement: (1.2 MB) PDF [file ehp.1409049.s001.acco.pdf]

Environ Health Perspect
doi: 10.1289/ehp.1409049

Note to Readers: *EHP* strives to ensure that all journal content is accessible to all readers. However, some figures and Supplemental Material published in *EHP* articles may not conform to 508 standards due to the complexity of the information being presented. If you need assistance accessing journal content, please contact ehp508@niehs.nih.gov. Our staff will work with you to assess and meet your accessibility needs within 3 working days.

Supplemental Material

Exposure to Endocrine-Disrupting Chemicals during Pregnancy and Weight at 7 Years of Age: A Multi-pollutant Approach

Keren Agay-Shay, David Martinez, Damaskini Valvi , Raquel Garcia-Esteban, Xavier Basagaña, Oliver Robinson, Maribel Casas, Jordi Sunyer, and Martine Vrijheid

Table of Contents

Description of the Imputation Procedure

Table S1: Crude and adjusted associations (beta coefficient, 95% CI) between maternal exposure to tertiles of 27 EDCs and child zBMI at age 7 years, single pollutant models, for complete case and imputed data (n=470).

Table S2: Summery of the Factors Loading for Four Factors Using Principle Component Analysis with Varimax Rotation.

Figure S1. Missing patterns of the 27 EDCs and 470 participants. All 27 EDCs variables had missing values. Only 40 participants had complete data without any missing value. Out of 12,690 values, 28.7% had a complete data. There were 74 different patterns of missingness for the 27 exposures. Abbreviations: As: Arsenic, BDEs: polybrominated diphenyl ethers congeners, bHCH: beta-hexachlorohexane, BPA: bisphenol A, Cd: Cadmium, DDE: dichlorodi-phenyldichloroethylene, DEHP: di-2-ethylhexyl phthalate, DEP: di-ethyl phthalate,

doi: 10.1289/ehp.1409049

DINP: di-isononyl phthalate, DnBP: di-n-butyl phthalate, EDCs: endocrine disrupting chemical, HCB:hexachlorobenzene, Hg: Mercury, MBzP: mono-benzyl phthalate, MEHHP: mono-(2-ethyl-5-hydroxyhexyl) phthalate, MEHP: mono-(2-ethylhexyl) phthalate, MEOHP: mono-(2-ethyl-5-oxohexyl) phthalate, MEP: mono-ethyl phthalate, MiBP: mono-iso-butyl phthalate, MnBP: mono-n-butyl phthalate, Pb: Lead, PCB: polychlorinated biphenyls congeners, p_2cxMMHP/ MCMHP: mono-(2-carboxyhexyl) phthalate, p_5cxMEPP/ MECPP: mono(2-ethyl-5-carboxypentyl), p_7OHMMeOP: mono(4-methyl-7-hydroxyoctyl).

Figure S2. Pearson correlation coefficients matrix of the 27 EDCs exposures. Abbreviations: As: Arsenic, bHCH: beta-hexachlorohexane, BPA: bisphenol A, Cd: Cadmium, DDE: dichlorodi-phenyldichloroethylene, EDCs: endocrine disrupting chemical, HCB :hexachlorobenzene, Hg: Mercury, MBzP: mono-benzyl phthalate, MCMHP : mono-(2-carboxyhexyl) phthalate, MECPP: mono(2-ethyl-5-carboxypentyl), MEHHP: mono-(2-ethyl-5-hydroxyhexyl) phthalate, MEHP: mono-(2-ethylhexyl) phthalate, MEOHP: mono-(2-ethyl-5-oxohexyl) phthalate, MEP: mono-ethyl phthalate, MiBP: mono-iso-butyl phthalate, MnBP: mono-n-butyl phthalate, Pb: Lead, PBDEs: polybrominated diphenyl ethers congeners, PCBs :polychlorinated biphenyls congeners, X_7OHMMeOP: mono(4-methyl-7-hydroxyoctyl).

Figure S3. Crude and adjusted association between maternal exposure to tertiles of 27 EDCs and risk of overweight child (relative risks (RR), 95% CI) at age 7 years using, a single pollutant models, for complete case and imputed data (n=470). Abbreviations: As: Arsenic, BDE: polybrominated diphenyl ethers congeners, bHCH: beta-hexachlorohexane, BPA: bisphenol A, Cd: Cadmium, CI: confidence interval, DDE: dichlorodi-phenyldichloroethylene, EDCs: endocrine disrupting chemical, HCB :hexachlorobenzene, Hg: Mercury, MBzP: mono-benzyl phthalate, MCMHP : mono-(2-carboxyhexyl) phthalate, MECPP: mono(2-ethyl-5-carboxypentyl), MEHHP: mono-(2-ethyl-5-hydroxyhexyl) phthalate, MEHP: mono-(2-ethylhexyl) phthalate, MEOHP: mono-(2-ethyl-5-oxohexyl) phthalate, MEP: mono-ethyl phthalate, MiBP: mono-iso-butyl phthalate, MnBP: mono-n-butyl phthalate, Pb: Lead, sPCB :polychlorinated biphenyls congeners, 7OHMMeOP: mono(4-methyl-7-hydroxyoctyl).

doi: 10.1289/ehp.1409049

Description of the Imputation Procedure

• Software used and key setting: STATA 12.1 software (Stata Corporation, College Station, Texas) –ice command (with 10 cycles).

• Number of imputed datasets created: 100.

• Variables included in the imputation procedure:

- Child variables:

sex, season at last menstrual period, gestational age, birth weight and length, small for gestational age, type of delivery, duration of predominant breastfeeding and any breastfeeding;

at child age 14 months: exact age, waist to height ratio, zBMI

at child age 4 years: exact age, waist to height ratio, zBMI, sedentary behavior during week and during weekend, sleeping habits, physical activity of the child, nutritional behavior (total caloric, protein intake, carbohydrate intake, total fat intake)

at child age 7 years: exact age, waist to height ratio, zBMI, sedentary behavior during week and during weekend, sleeping habits

- Parental variables:

during pregnancy: maternal and paternal age, maternal and paternal origin country (Spain), maternal and paternal BMI, maternal and paternal social class, maternal and paternal education, maternal and paternal smoking, maternal weight gain, METS, maternal nutritional behavior (total caloric, protein intake, carbohydrate intake, total fat intake, sugar intake, fruits and vegetables intake, any alcohol consumption), dogs at home, maternal thyroid hormone levels (TSH, FT4 and TT3), maternal chemicals levels: levels of urine average 1 and 3 trimester phthalates creatinine adjusted [MEP, MnBP, MiBP, MBzP, 7OH-MMeOP, MECPP, MEHHP, MEOHP, MEHP,MCMHP], level of urine average 1 and 3 trimester BPA creatinine adjusted , levels of urine average 1 and 3

trimester metals creatinine adjusted [Cd, As, Pb] levels of cord blood Hg, levels of lipids adjusted organochlorine measured in serum [DDE, HCB, bHCH, PCBs (138, 153, 180)] levels of PBDEs measured in colostrum[47, 99, 100, 153, 154, 209], levels of organochlorine measured in colostrum[DDE, DDT,HCB, bHCH, PCBs (138, 153, 180)], levels of cotininine creatinine adjusted;

at child age 14 months: maternal and paternal smoking, dogs at home;

at child age 4 years: maternal and paternal smoking, dogs at home;

at child age 7 years: maternal and paternal smoking.

All variables were included as continuous variables except the following:

- Dichotomous variables:

Child variables: sex, small for gestational age, physical activity of the child.

Parental variables:

during pregnancy: maternal and paternal origin country (Spain), maternal and paternal smoking, any alcohol consumption, dogs at home

at child age 14 months: maternal and paternal smoking, dogs at home;

at child age 4 years: maternal and paternal smoking, dogs at home;

at child age 7 years: maternal and paternal smoking.

- Categorical variables (all categories were included):

Child variable: season at last menstrual period (4 categories), type of delivery (3 categories), duration of predominant breastfeeding and any breastfeeding (4 categories), sedentary behavior during week and during weekend at child age 4 years and age 7 years (3 categories), sleeping time at child age 4 years (4 categories), sleeping time at age 7 years(3 categories)

doi: 10.1289/ehp.1409049

Parental variables:

during pregnancy: maternal and paternal social class(3 categories), maternal and paternal education(3 categories), maternal weight gain(3 categories)

• Treatment of non-normally distributed variables: log-transformed.

• Treatment of binary/categorical variables: logistic, ordinal, and multinomial models.

doi: 10.1289/ehp.1409049

Table S1: Crude and adjusted associations (beta coefficient, 95% CI) between maternal exposure to tertiles of 27 EDCs and child zBMI at age 7 years, single pollutant models, for complete case and imputed data (n=470).

Exposure	Crude complete case β (95% CI)	Crude Imputed β (95% CI)	Adjusted complete case β (95% CI)	Adjusted Imputed β (95% CI)
MEP				
1	Reference	Reference	Reference	Reference
2	0.16 (-0.15, 0.47)	0.17 (-0.15, 0.48)	0.09 (-0.21, 0.38)	0.12 (-0.17, 0.41)
3	0.23 (-0.08, 0.54)	0.26 (-0.06, 0.58)	0.11 (-0.18, 0.41)	0.20 (-0.1, 0.50)
MnBP				
1	Reference	Reference	Reference	Reference
3	0.03 (-0.28, 0.34)	0.01 (-0.3, 0.31)	0.12 (-0.17, 0.42)	0.03 (-0.26, 0.31)
3	0.00 (-0.31, 0.31)	-0.04 (-0.35, 0.27)	-0.08 (-0.37, 0.22)	-0.11 (-0.4, 0.18)
MiBP				
1	Reference	Reference	Reference	Reference
2	0.13 (-0.18, 0.44)	0.11 (-0.2, 0.43)	0.21 (-0.09, 0.51)	0.1 (-0.2, 0.39)
3	-0.05 (-0.36, 0.26)	-0.07 (-0.38, 0.25)	0.06 (-0.23, 0.35)	-0.05 (-0.35, 0.26)
MBzP				
1	Reference	Reference	Reference	Reference
2	0.03 (-0.28, 0.34)	0.02 (-0.29, 0.33)	0.06 (-0.23, 0.36)	0.02 (-0.27, 0.30)
3	-0.07 (-0.38, 0.25)	-0.03 (-0.35, 0.29)	-0.1 (-0.4, 0.19)	-0.05 (-0.35, 0.24)
7OHMMeOP				
1	Reference	Reference	Reference	Reference
2	-0.22 (-0.54, 0.09)	-0.18 (-0.5, 0.13)	-0.21 (-0.51, 0.09)	-0.16 (-0.46, 0.14)
3	-0.36 (-0.68, -0.05)	-0.32 (-0.64, 0)	-0.29 (-0.59, 0.01)	-0.27 (-0.57, 0.02)
MECPP				
1	Reference	Reference	Reference	Reference
2	-0.13 (-0.44, 0.18)	-0.13 (-0.43, 0.18)	-0.21 (-0.51, 0.08)	-0.15 (-0.44, 0.14)
3	-0.07 (-0.38, 0.25)	-0.05 (-0.37, 0.27)	-0.26 (-0.55, 0.04)	-0.15 (-0.45, 0.14)
MEHHP				
1	Reference	Reference	Reference	Reference
2	0.07 (-0.24, 0.38)	0.06 (-0.26, 0.37)	-0.08 (-0.38, 0.21)	0 (-0.29, 0.29)
3	-0.04 (-0.35, 0.27)	-0.05 (-0.36, 0.27)	-0.09 (-0.39, 0.21)	-0.08 (-0.37, 0.21)
MEOHP				
1	Reference	Reference	Reference	Reference
2	0.09 (-0.23, 0.4)	0.06 (-0.25, 0.38)	-0.12 (-0.42, 0.17)	-0.02 (-0.32, 0.27)
3	0.01 (-0.3, 0.32)	0.04 (-0.28, 0.36)	-0.14 (-0.44, 0.16)	-0.05 (-0.35, 0.25)
MEHP				
1	Reference	Reference	Reference	Reference
2	0.06 (-0.25, 0.37)	0.08 (-0.24, 0.39)	0.05 (-0.25, 0.34)	0.11 (-0.18, 0.39)
3	-0.12 (-0.43, 0.19)	-0.12 (-0.43, 0.19)	-0.03 (-0.32, 0.27)	-0.11 (-0.39, 0.18)
MCMHP				
1	Reference	Reference	Reference	Reference
2	0.06 (-0.3, 0.42)	0.07 (-0.26, 0.41)	-0.09 (-0.43, 0.25)	0 (-0.31, 0.31)
3	0.03 (-0.33, 0.39)	0.02 (-0.31, 0.36)	-0.14 (-0.49, 0.2)	-0.05 (-0.36, 0.26)
BPA				
1	Reference	Reference	Reference	Reference
2	0.07 (-0.24, 0.38)	0.06 (-0.25, 0.36)	-0.01 (-0.31, 0.28)	0.01 (-0.28, 0.29)
3	0.08 (-0.23, 0.39)	0.1 (-0.2, 0.41)	-0.06 (-0.35, 0.24)	0.03 (-0.25, 0.32)
Pb				

doi: 10.1289/ehp.1409049

Exposure	Crude complete case β (95% CI)	Crude Imputed β (95% CI)	Adjusted complete case β (95% CI)	Adjusted Imputed β (95% CI)
1	Reference	Reference	Reference	Reference
2	0.19 (-0.16, 0.54)	0.12 (-0.21, 0.45)	0.26 (-0.07, 0.59)	0.18 (-0.13, 0.49)
3	-0.02 (-0.37, 0.33)	-0.12 (-0.46, 0.21)	0.08 (-0.27, 0.44)	-0.06 (-0.4, 0.28)
Cd				
1	Reference	Reference	Reference	Reference
2	-0.04 (-0.4, 0.32)	0.11 (-0.25, 0.46)	-0.08 (-0.42, 0.26)	0.1 (-0.24, 0.44)
3	-0.11 (-0.47, 0.25)	0.01 (-0.35, 0.36)	-0.11 (-0.46, 0.24)	0.01 (-0.32, 0.35)
As				
1	Reference	Reference	Reference	Reference
2	-0.01 (-0.32, 0.3)	-0.01 (-0.31, 0.29)	-0.08 (-0.37, 0.21)	-0.01 (-0.29, 0.26)
3	-0.09 (-0.4, 0.22)	-0.07 (-0.38, 0.23)	-0.08 (-0.37, 0.21)	-0.05 (-0.34, 0.24)
Hg				
1	Reference	Reference	Reference	Reference
2	-0.08 (-0.41, 0.24)	-0.1 (-0.41, 0.21)	0.03 (-0.28, 0.35)	-0.04 (-0.33, 0.26)
3	-0.07 (-0.4, 0.25)	-0.09 (-0.39, 0.22)	0.05 (-0.28, 0.38)	0.01 (-0.28, 0.3)
DDE				
1	Reference	Reference	Reference	Reference
2	0.14 (-0.14, 0.43)	0.12 (-0.16, 0.4)	0.16 (-0.11, 0.44)	0.08 (-0.18, 0.35)
3	0.16 (-0.12, 0.45)	0.17 (-0.11, 0.45)	0.27 (-0.02, 0.56)	0.19 (-0.09, 0.48)
HCB				
1	Reference	Reference	Reference	Reference
2	0.18 (-0.1, 0.46)	0.19 (-0.09, 0.46)	0.18 (-0.11, 0.47)	0.19 (-0.08, 0.47)
3	0.46 (0.18, 0.74)	0.47 (0.19, 0.75)	0.49 (0.16, 0.82)	0.44 (0.13, 0.75)
βHCH				
1	Reference	Reference	Reference	Reference
2	0.21 (-0.07, 0.49)	0.21 (-0.07, 0.49)	0.2 (-0.08, 0.47)	0.21 (-0.05, 0.48)
3	0.43 (0.15, 0.71)	0.41 (0.13, 0.69)	0.37 (0.08, 0.67)	0.32 (0.04, 0.61)
PCB138				
1	Reference	Reference	Reference	Reference
2	-0.09 (-0.37, 0.19)	-0.09 (-0.37, 0.19)	0.13 (-0.17, 0.42)	0.14 (-0.14, 0.43)
3	-0.1 (-0.38, 0.18)	-0.09 (-0.37, 0.19)	0.36 (0.04, 0.68)	0.31 (0, 0.62)
PCB153				
1	Reference	Reference	Reference	Reference
2	-0.32 (-0.6, -0.04)	-0.3 (-0.58, -0.02)	-0.13 (-0.42, 0.17)	-0.1 (-0.38, 0.19)
3	-0.24 (-0.52, 0.04)	-0.24 (-0.51, 0.04)	0.18 (-0.16, 0.52)	0.15 (-0.17, 0.48)
PCB180				
1	Reference	Reference	Reference	Reference
2	-0.3 (-0.58, -0.02)	-0.28 (-0.56, 0)	0.05 (-0.25, 0.36)	0.06 (-0.24, 0.36)
3	-0.19 (-0.47, 0.09)	-0.19 (-0.47, 0.09)	0.41 (0.05, 0.77)	0.35 (0, 0.71)
PBDE47				
1	Reference	Reference	Reference	Reference
2	0 (-0.42, 0.43)	-0.04 (-0.41, 0.33)	0.02 (-0.4, 0.43)	-0.02 (-0.36, 0.33)
3	-0.05 (-0.48, 0.38)	-0.12 (-0.52, 0.29)	0.05 (-0.37, 0.46)	-0.04 (-0.42, 0.35)
PBDE99				
1	Reference	Reference	Reference	Reference
2	0.02 (-0.4, 0.45)	-0.04 (-0.4, 0.33)	-0.08 (-0.49, 0.34)	-0.05 (-0.39, 0.29)
3	0 (-0.43, 0.43)	-0.05 (-0.43, 0.34)	0.03 (-0.39, 0.44)	-0.01 (-0.36, 0.35)
PBDE100				
1	Reference	Reference	Reference	Reference
2	-0.02 (-0.45, 0.4)	-0.14 (-0.52, 0.23)	-0.11 (-0.53, 0.32)	-0.1 (-0.46, 0.25)

doi: 10.1289/ehp.1409049

Exposure	Crude complete case β (95% CI)	Crude Imputed β (95% CI)	Adjusted complete case β (95% CI)	Adjusted Imputed β (95% CI)
3	-0.27 (-0.7, 0.16)	-0.35 (-0.74, 0.03)	-0.16 (-0.57, 0.26)	-0.27 (-0.63, 0.09)
PBDE153				
1	Reference	Reference	Reference	Reference
2	-0.39 (-0.82, 0.03)	-0.15 (-0.52, 0.21)	-0.31 (-0.73, 0.11)	-0.09 (-0.44, 0.27)
3	-0.24 (-0.66, 0.19)	-0.15 (-0.54, 0.24)	-0.05 (-0.48, 0.37)	-0.01 (-0.39, 0.36)
PBDE154				
1	Reference	Reference	Reference	Reference
2	-0.39 (-0.82, 0.03)	-0.13 (-0.5, 0.23)	-0.31 (-0.73, 0.11)	-0.07 (-0.43, 0.28)
3	-0.24 (-0.66, 0.19)	-0.16 (-0.55, 0.23)	-0.05 (-0.48, 0.37)	-0.01 (-0.38, 0.36)
PBDE209				
1	Reference	Reference	Reference	Reference
2	0.02 (-0.41, 0.44)	0 (-0.4, 0.41)	0.15 (-0.27, 0.56)	0.06 (-0.31, 0.42)
3	-0.32 (-0.74, 0.1)	-0.12 (-0.52, 0.28)	-0.16 (-0.57, 0.25)	-0.04 (-0.41, 0.33)

Abbreviations :As: Arsenic, βHCH: beta-hexachlorohexane, BPA: bisphenol A, Cd: Cadmium, CI: confidence interval, DDE: dichloro-diphenyldichloroethylene, EDCs: endocrine disrupting chemical, , HCB: hexachlorobenzene, Hg: Mercury MBzP: mono-benzyl phthalate, MEHHP: mono-(2-ethyl-5-hydroxyhexyl) phthalate, MEHP: mono-(2-ethylhexyl) phthalate, MEOHP: mono-(2-ethyl-5-oxohexyl) phthalate, MEP: mono-ethyl phthalate, MiBP: mono-iso-butyl phthalate, MnBP: mono-n-butyl phthalate, Pb: Lead, PBDEs: poly-brominated diphenyl ethers, PCBs: polychlorinated biphenyls, MCMHP : mono-(2-carboxyhexyl) phthalate, MECPP: mono(2-ethyl-5-carboxypentyl), 7OHMMeOP: mono(4-methyl-7-hydroxyoctyl), zBMI: body mass index z-score

Table S2: Summery of the Factors Loading for Four Factors Using Principle Component Analysis with Varimax Rotation.

**EDCs**	**Factor 1 PBDEs**	**Factor2 Phthalates**	**Factor3 Organochlorines**	**Factor4 MEP, As, Hg, BPA, PBDE153, PBDE154 [a]**	**Unexplained**
**MEP**	0.01	0.12	-0.04	**0.32**	0.14
**MnBP**	0.03	**0.25**	0.00	-0.07	0.08
**MiBP**	0.03	**0.21**	-0.08	0.01	0.06
**MBzP**	0.03	**0.28**	-0.04	0.06	0.09
**7OHMMeOP**	0.00	**0.31**	-0.04	0.15	0.08
**MECPP**	0.00	**0.37**	0.03	-0.03	0.03
**MEHHP**	0.01	**0.41**	0.02	-0.03	0.03
**MEOHP**	0.00	**0.39**	0.03	-0.03	0.02
**MEHP**	0.00	**0.32**	0.01	0.00	0.05
**MCMHP**	0.00	**0.31**	0.01	-0.01	0.03
**BPA**	-0.03	0.10	-0.11	**0.23**	0.08
**Cd**	-0.03	0.12	0.07	0.06	0.08
**As**	-0.07	0.00	-0.01	**0.67**	0.07
**Pb**	-0.04	0.05	0.07	0.06	0.07
**Hg**	-0.01	-0.07	0.04	**0.31**	0.08
**DDE**	-0.05	0.08	**0.24**	**-0.24**	0.10
**HCB**	0.00	0.00	**0.51**	-0.03	0.04
**βHCH**	-0.03	0.04	**0.45**	**-0.21**	0.04
**PCB138**	-0.01	0.00	**0.36**	0.11	0.03
**PCB153**	-0.01	-0.01	**0.38**	0.14	0.02
**PCB180**	-0.01	-0.02	**0.37**	0.14	0.03
**PBDE47**	**0.55**	0.02	-0.08	-0.08	0.08
**PBDE99**	**0.55**	0.02	0.03	-0.08	0.08
**PBDE100**	**0.37**	0.00	-0.03	-0.01	0.10
**PBDE153**	**0.31**	-0.06	0.12	**0.23**	0.08
**PBDE154**	**0.31**	-0.06	0.12	**0.21**	0.08
**PBDE209**	**0.23**	0.01	0.03	-0.01	0.10

Abbreviations :As: Arsenic, βHCH: beta-hexachlorohexane, BPA: bisphenol A, Cd: Cadmium, CI: confidence interval, DDE: dichloro-diphenyldichloroethylene, EDCs: endocrine disrupting chemical, , HCB: hexachlorobenzene, Hg: Mercury MBzP: mono-benzyl phthalate, MEHHP: mono-(2-ethyl-5-hydroxyhexyl) phthalate, MEHP: mono-(2-ethylhexyl) phthalate, MEOHP: mono-(2-ethyl-5-oxohexyl) phthalate, MEP: mono-ethyl phthalate, MiBP: mono-iso-butyl phthalate, MnBP: mono-n-butyl phthalate, Pb: Lead, PBDEs: poly-brominated diphenyl ethers, PCBs: polychlorinated biphenyls, MCMHP : mono-

doi: 10.1289/ehp.1409049

(2-carboxyhexyl) phthalate, MECPP: mono(2-ethyl-5-carboxypentyl), 7OHMMeOP: mono(4-methyl-7-hydroxyoctyl)

[a] Factor 4 loaded with: MEP, As, Hg, BPA, PBDE153 ,PBDE154, DDE and βHCH had negative loading values.

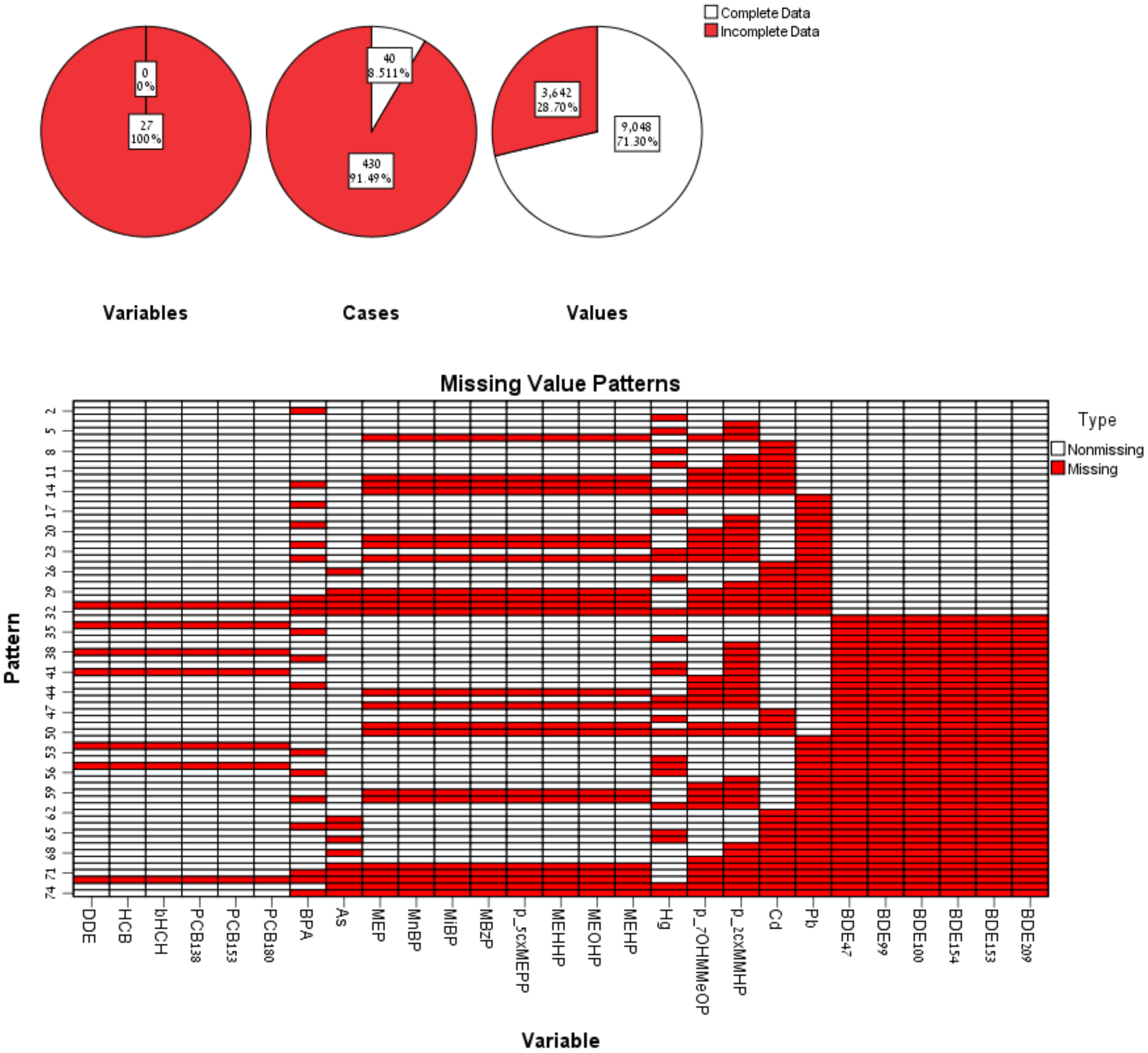

Figure S1. Missing patterns of the 27 EDCs and 470 participants. All 27 EDCs variables had missing values. Only 40 participants had complete data without any missing value. Out of 12,690 values, 28.7% had a complete data. There were 74 different patterns of missingness for the 27 exposures. Abbreviations: As: Arsenic, BDEs: polybrominated diphenyl ethers congeners, bHCH: beta-hexachlorohexane, BPA: bisphenol A, Cd: Cadmium, DDE: dichlorodi-phenyldichloroethylene, DEHP: di-2-ethylhexyl phthalate, DEP: di-ethyl phthalate, DINP: di-isononyl phthalate, DnBP: di-n-butyl phthalate, EDCs: endocrine disrupting chemical, HCB:hexachlorobenzene, Hg: Mercury, MBzP: mono-benzyl phthalate, MEHHP: mono-(2-ethyl-5-hydroxyhexyl) phthalate, MEHP: mono-(2-ethylhexyl) phthalate, MEOHP: mono-(2-

doi: 10.1289/ehp.1409049

ethyl-5-oxohexyl) phthalate, MEP: mono-ethyl phthalate, MiBP: mono-iso-butyl phthalate, MnBP: mono-n-butyl phthalate, Pb: Lead, PCB: polychlorinated biphenyls congeners, p_**2**cxMMHP/ MCMHP: mono-(2-carboxyhexyl) phthalate, p_**5**cxMEPP/ MECPP: mono(2-ethyl-5-carboxypentyl), p_7OHMMeOP: mono(4-methyl-7-hydroxyoctyl)

Environ Health Perspect
doi: 10.1289/ehp.1409049

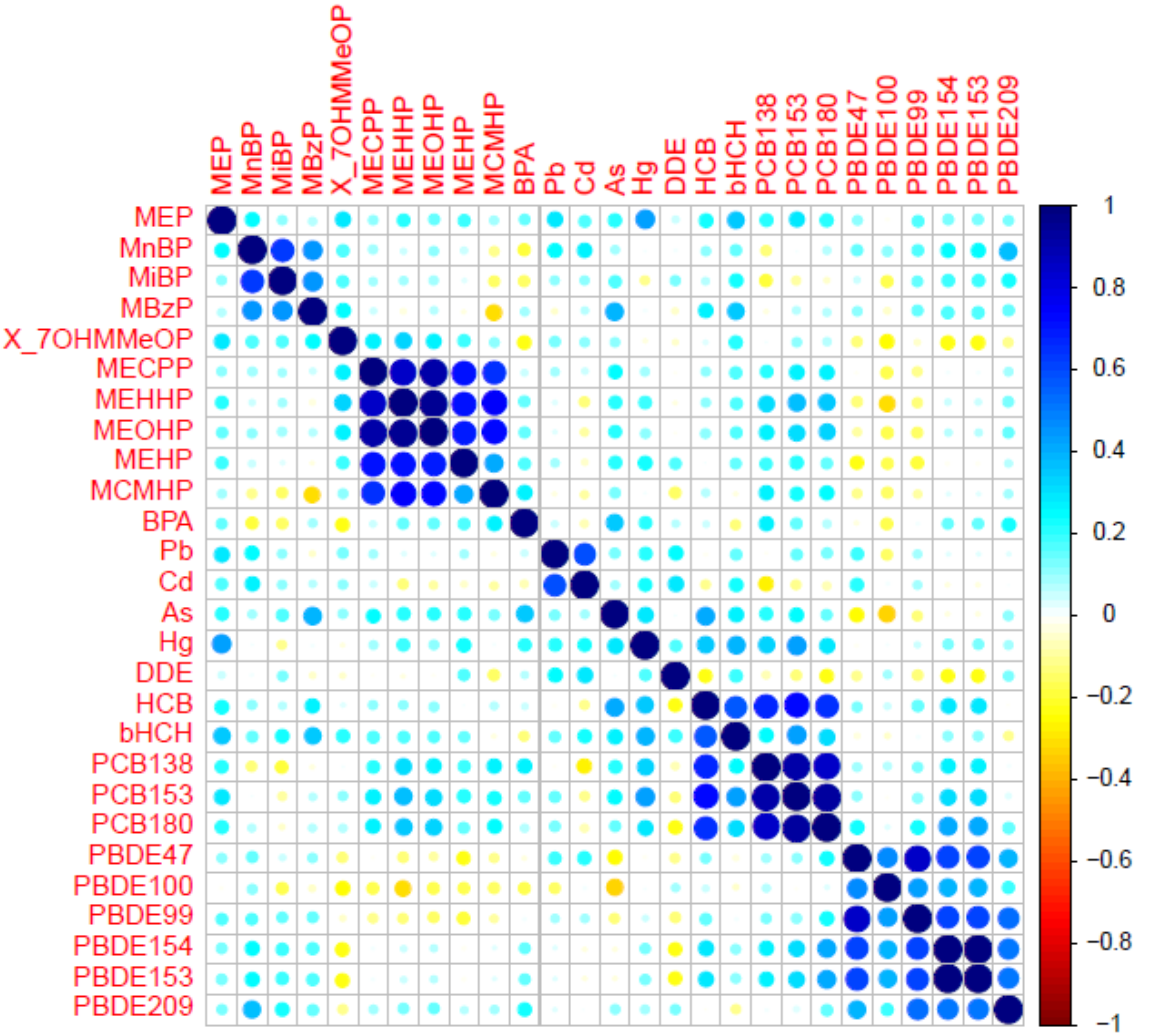

Figure S2. Pearson correlation coefficients matrix of the 27 EDCs exposures. Abbreviations: As: Arsenic, bHCH: beta-hexachlorohexane, BPA: bisphenol A, Cd: Cadmium, DDE: dichlorodi-phenyldichloroethylene, EDCs: endocrine disrupting chemical, HCB :hexachlorobenzene, Hg: Mercury, MBzP: mono-benzyl phthalate, MCMHP : mono-(2-carboxyhexyl) phthalate, MECPP: mono(2-ethyl-5-carboxypentyl), MEHHP: mono-(2-ethyl-5-hydroxyhexyl) phthalate, MEHP: mono-(2-ethylhexyl) phthalate, MEOHP: mono-(2-ethyl-5-oxohexyl) phthalate, MEP: mono-ethyl phthalate, MiBP: mono-iso-butyl phthalate, MnBP: mono-n-butyl phthalate, Pb: Lead, PBDEs: polybrominated diphenyl ethers congeners, PCBs :polychlorinated biphenyls congeners, X_7OHMMeOP: mono(4-methyl-7-hydroxyoctyl).

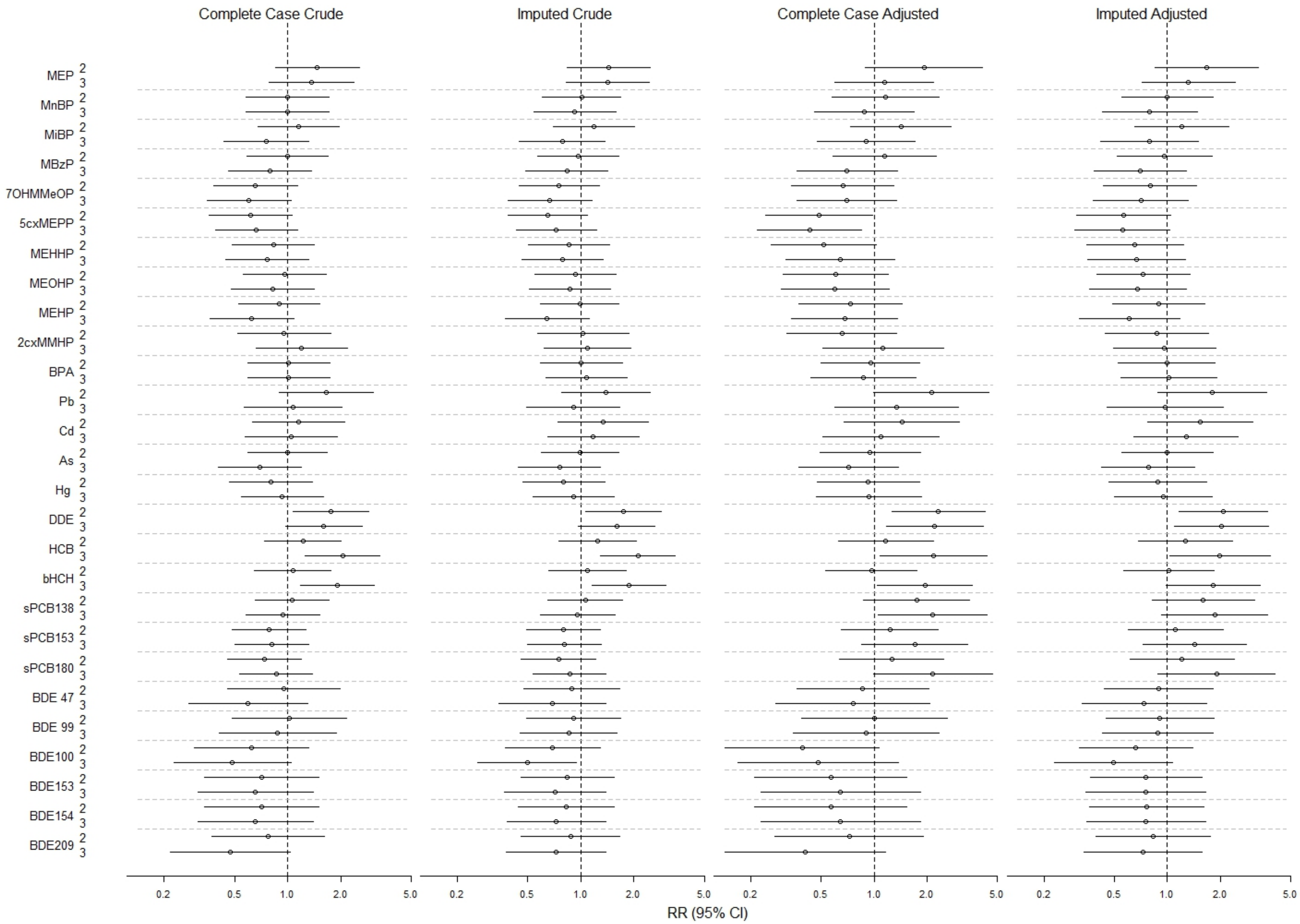
Complete Case Crude
Imputed Crude
Complete Case Adjusted
Imputed Adjusted
MEP
MnBP
MiBP
MBzP
7OHMMeOP
5cxMEPP
MEHHP
MEOHP
MEHP
2cxMMHP
BPA
Pb
Cd
As
Hg
DDE
HCB
bHCH
sPCB138
sPCB153
sPCB180
BDE 47
BDE 99
BDE100
BDE153
BDE154
BDE209
0.2
0.5
1.0
2.0
5.0
RR (95% CI)

Figure S3. Crude and adjusted association between maternal exposure to tertiles of 27 EDCs and risk of overweight child (relative risks (RR), 95% CI) at age 7 years using, a single pollutant models, for complete case and imputed data (n=470). Abbreviations: As: Arsenic, BDE: polybrominated diphenyl ethers congeners, bHCH: beta-hexachlorohexane, BPA: bisphenol A, Cd: Cadmium, CI: confidence interval, DDE: dichlorodi-phenyldichloroethylene, EDCs: endocrine disrupting chemical, HCB :hexachlorobenzene, Hg: Mercury, MBzP: mono-benzyl phthalate, MCMHP : mono-(2-carboxyhexyl) phthalate, MECPP: mono(2-ethyl-5-carboxypentyl), MEHHP: mono-(2-ethyl-5-hydroxyhexyl) phthalate, MEHP: mono-(2-ethylhexyl) phthalate, MEOHP: mono-(2-ethyl-5-oxohexyl) phthalate, MEP: mono-ethyl phthalate, MiBP: mono-iso-butyl phthalate, MnBP: mono-n-butyl phthalate, Pb: Lead, sPCB :polychlorinated biphenyls congeners, 7OHMMeOP: mono(4-methyl-7-hydroxyoctyl).
